# Impact of social determinants of health on anticoagulant use among patients with atrial fibrillation: Systemic review and meta-analysis

**DOI:** 10.1097/MD.0000000000029997

**Published:** 2022-09-02

**Authors:** Rasha Khatib, Nicole Glowacki, John Byrne, Peter Brady

**Affiliations:** a Advocate Aurora Research Institute, Advocate Aurora Health, Downers Grove, IL; b School of Molecular & Cellular Biology, University of Illinois at Urbana-Champaign, Urbana, IL; c Department of Cardiovascular Medicine, Advocate Illinois Masonic Medical Center, Chicago, IL.

**Keywords:** anticoagulant, atrial fibrillation, health disparities, social determinants of health, stroke

## Abstract

A growing body of literature now exists examining associations between social determinants of health (SDOH) and adverse outcomes in patients with atrial fibrillation; however, little is available on anticoagulant prescriptions and the impact of SDOH.

**Purpose:**

Evaluate the impact of SDOH on anticoagulant prescriptions in patients with atrial fibrillation.

**Data Sources:**

Medline and Embase databases up to January 2021.

**Study Selection:**

Noninterventional studies were included if they reported associations between at least 1 of 14 SDOH domains and anticoagulant prescription in patients with atrial fibrillation. Two investigators independently screened and collected data.

**Data Extraction:**

Two investigators independently screened and collected data.

**Data Synthesis:**

Meta-analyses using random-effect models evaluated associations between SDOH and receiving an anticoagulant prescription. We included 13 studies, 11 of which were included in meta-analyses that reported on the impact of 9 of the 14 SDOH included in the search. Pooled estimates indicate a 0.85 (95% confidence interval [CI]: 0.75, 0.97) lower odds of receiving anticoagulant prescriptions among Black compared to non-Black patients (reported in 6 studies); 0.42 (95% CI: 0.32, 0.55) lower odds of receiving anticoagulant prescriptions among patients with mental illness compared to those without mental illness (2 studies); and a 0.64 (95% CI: 0.42, 0.96) lower likelihood of receiving oral anticoagulant prescription among employed patients compared to unemployed patients (2 studies).

**Limitations:**

SDOH lack consistent definitions and measures within the electronic health record.

**Conclusion:**

The literature reports on only half of the SDOH domains we searched for, indicating that many SDOH are not routinely assessed. Second, social needs impact the decision to prescribe anticoagulants, confirming the need to screen for and address social needs in the clinical setting to support clinicians in providing guideline concordant care to their patients.

**Registration:**

This systematic review and meta-analysis was registered with PROSPERO.

## 1. Introduction

Atrial fibrillation is estimated to affect 2.7 to 6.1 million people in the Unites States and is associated with increased risk of stroke, heart failure, and death.^[1,2]^ Anticoagulant therapy is indicated for patients with atrial fibrillation and is effective and safe in preventing thromboembolic events.^[3]^ Despite the evidence, there are reports describing suboptimal anticoagulant treatment among patients with an atrial fibrillation diagnosis.^[4,5]^ Factors that impact anticoagulant treatment are multifaceted and are likely impacted by social determinants of health (SDOH) which are defined as “the conditions in which people are born, grow, work, live, age, and the wider set of forces and systems shaping the conditions of daily life.”^[6]^

There is a growing body of literature examining the association between SDOH domains and cardiovascular and other chronic diseases, especially in the United States given the observed disparities in morbidity and mortality.^[7,8]^ However, the methodology and quality of the literature vary. Further, despite consensus that SDOH are pivotal in understanding health outcome inequities, the definitions and inclusion of specific domains of SDOH vary across several key organizations.^[9–11]^

Currently, a comprehensive review of SDOH domains that have been explored in the literature and their associations with use of anticoagulants for patients diagnosed with atrial fibrillation does not exist. We identified a list of SDOH a priori based on a review developed by the US Preventive Service Task Force that identifies a comprehensive list of key domains for SDOH compiled from key organizations that have contributed to the literature in addressing the effects of SDOH on health and wellbeing in the US population.^[12]^ We use this framework to systematically review the literature to evaluate associations between SDOH and anticoagulant use among patients recently diagnosed with atrial fibrillation. The review is reported according to the Preferred Reporting Items for Systematic Reviews and Meta-Analyses (PRISMA) guidelines.^[13]^

## 2. Methods

### 2.1. Data sources and search

We searched MEDLINE through PubMed (January 1996 to January 5, 2021) and EMBASE (1974 to January 5, 2021) (Supplemental Digital Content 1 and 2, http://links.lww.com/MD/G992). The search strategy consisted of predefined keywords specific to each database. The key words included “Atrial Fibrillation” AND terms for each pre identified social determinant of health AND “anticoagulants.” Additionally, reference lists of relevant studies and systematic reviews were scanned, and clinical experts in the field of anticoagulation management were consulted for additional references. We used Epistemonikos (www.epistemonikos.org) to identify relevant published systematic reviews and screened references.

### 2.2. Study selection

Studies were eligible if they included adult patients (≥18 years of age), recent (within 1 year) atrial fibrillation diagnosis, did not receive an anticoagulant at baseline, conducted in the United States, and evaluated the impact of at least 1 SDOH on the primary outcome which is documentation of receiving a prescription for an anticoagulant (whether or not the patient filled the prescription is beyond the scope of this review). A list of 16 SDOH domains were identified based on a report developed by the US Preventive Service Task Force and encompasses domains from Health People 2020, Accountable Health Communities Model, Community Preventive Task Force, and Campbell and Cochrane Equity Methods Group.^[12]^ The domains included are housing, food security, transportation, socioeconomic status and financial strain, violence and interpersonal safety (including domestic abuse, elder abuse, and child maltreatment), employment, community and social connections, education, health behaviors (including substance use/abuse, physical activity, and health diet), mental health, disabilities, neighborhood and built environment, race/ethnicity, culture, religion, immigration status, and language, healthcare access and health literacy, law and justice system and incarceration, and gender and sexual orientation.

SDOH definitions are expected to be different across countries due to variations in care models, insurance, and payer structures. To limit heterogeneity across included studies, we included studies conducted in the United States only. Inclusion was limited to observational studies including prospective, retrospective, cohort, case-control, and cross-sectional methods. Intervention studies and studies that did not include primary data, including review studies were excluded. Studies not published in peer-reviewed journals were excluded. Studies published in languages other than English were also excluded.

The outcomes prioritized for this review included a prescription or use of oral anticoagulants, including warfarin and direct oral anticoagulants (DOACs), specifically apixaban, rivaroxaban, dabigatran, or edoxaban.

The protocol for this review is registered in PROSPERO (CRD42021232333).

### 2.3. Data extraction and quality assessment

Two reviewers (JB and NG) independently screened titles, abstracts, and the full text of relevant articles. Based on prespecified inclusion and exclusion criteria, disagreement was resolved by consensus by a third reviewer (RK) when needed. One reviewer extracted data from each eligible study using a pretested data abstraction form, and data were checked by another reviewer to assess accuracy. Disagreements were resolved by discussion, and by a third reviewer when needed. The data collected included study and patient characteristics (study type, sample size, mean age, and proportion of females), inclusion criteria focusing on risk of stroke, SDOH examined, and outcomes. For each outcome of interest, the number of patients, number of events, odds ratios (ORs), and 95% confidence internals (CIs) were extracted. Variables adjusted for in the statistical models were also abstracted.

### 2.4. Risk of bias in individual studies

Risk of bias was assessed at the study level. Following the Cochrane Collaboration’s recommendation to present potential biases for each study instead of using scores to rate quality, a set of quality appraisal domains relevant to the type of studies included was applied.^[14]^ As recommended in the literature signaling questions were used to facilitate judgment about the risk of bias domains relevant to observational study methodology.^[15–17]^ Risk of bias for each domain was assessed qualitatively as “low risk” or “high risk.”^[15]^ Domains evaluated included bias due to confounding (were important confounding variables adjusted for), selection bias (was selection into the study unrelated to exposures and outcomes), information bias (were methods of outcome assessment comparable across exposure groups), and bias due to missing data (were reasons for missing data unrelated to exposure and outcomes). For bias due to confounding, a study was considered at high risk of bias if the effect estimate did not adjust for the following list of variables: age, sex, CHADS_2_ (or CHA_2_DS_2_-VASc), and bleeding risk.

### 2.5. Data synthesis and analysis

When applicable, estimates of effect, which included ORs across all included studies, and 95% CI were pooled for each social determinant of health evaluating anticoagulant prescription or use. Studies that included numbers or proportions only were included and proportions were converted to unadjusted ORs and bias due to confounding was reported as “high.” To allow for pooling estimates of effect when different references are used across included individual studies, some estimates were switched by taking the inverse of the estimates of individual studies and is noted in tables and figures in the results section.

Results were pooled if at least 2 studies reported the outcome of interest using the inverse variance approach and the random effects model. A random effects model was selected a priori for this meta- analysis due to expected heterogeneity across included studies.^[14]^ Heterogeneity was assessed using the I^2^ index and was deemed as moderate to high with an I^2^ over 50%.^[14]^ Subgroup analysis was conducted by type of anticoagulant among patients who received a prescription in each included study, subgroups included DOAC, warfarin, or both. Data were analyzed using RevMan 5.3. A narrative summary was created for studies that did not include enough information for a meta-analysis (eg, reported unadjusted OR only or did not report sample size). Results are reported separately for each SDOH domain as reported by the US Preventive Service Task Force.^[12]^

### 2.6. Ethical review

IRB approval was not obtained as data included in this study were retrieved from previously published studies in which IRB approval was obtained.

## 3. Results

### 3.1. Search results

**Figure 1.**
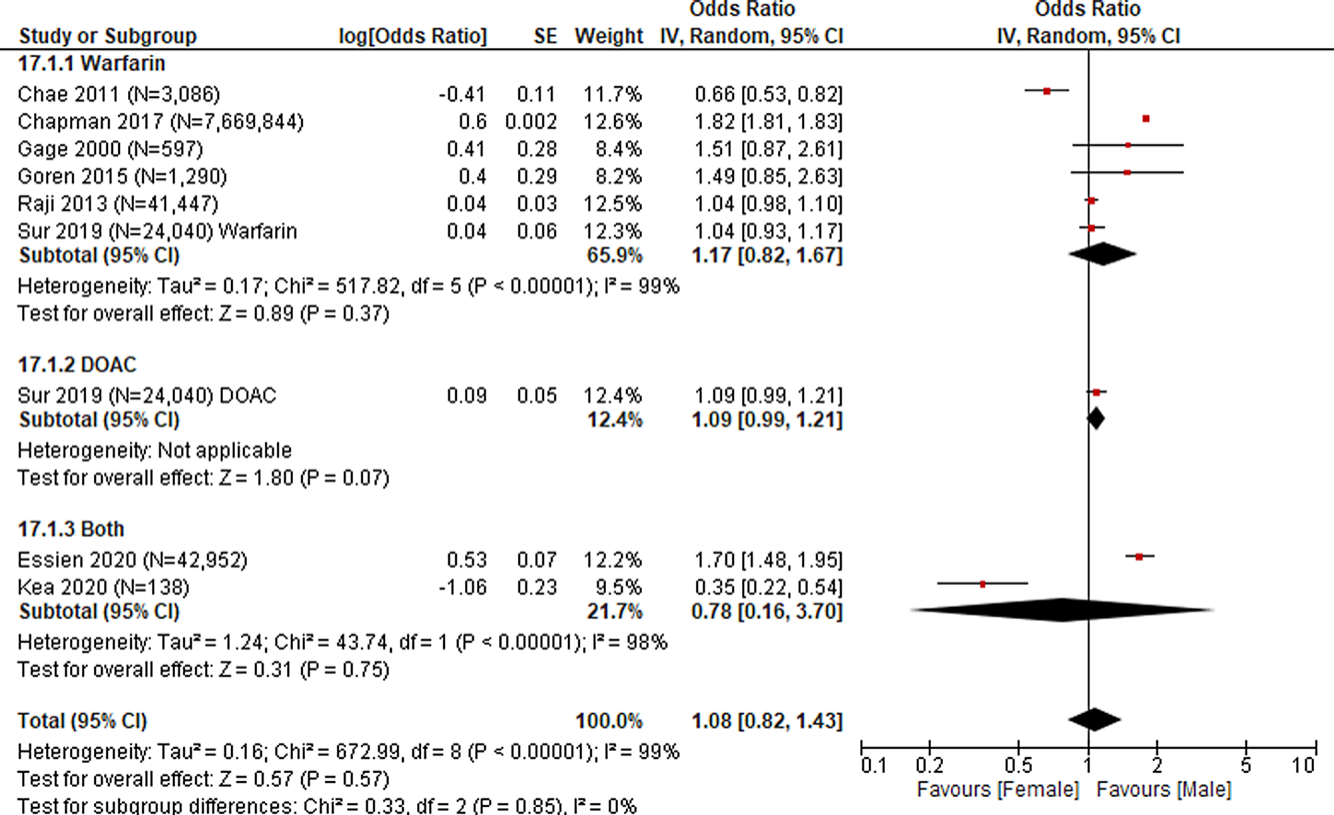
Sex – female (reference) versus male.

After excluding duplicates, a total of 3905 studies were screened for titles and abstract. Full text screening was conducted to exclude studies that are not eligible based on prespecified inclusion and exclusion criteria (eg, reviews, intervention studies, studies conducted outside of the United States, etc), leaving 100 for full text screening. After full text exclusions (Fig. 1), a total of 13 eligible studies were included in this systematic review, of which 10 were included in the meta-analyses.

### 3.2. Study characteristics

We identified 13 studies (total number of patients N = 7,906,445) that evaluated the impact of at least 1 social determinant of health on anticoagulant prescription or use. The number of patients per study ranged from to 138 to 7,669,844 patients (uses 2010 National Ambulatory Medical Care Survey [NAMCS)], a large publicly available database of patient records that are weighted to be representative of the US population). Table 1 presents study characteristics of included studies. The mean age ranged between 59 + 17.1 and 80 years (SD not reported). The proportion of females ranged between 2% and 72%. Source of data varied and included retrospective electronic health records^[20–23]^ (n = 4 studies), prospective registries^[24,25]^ (n = 2), national databases including veteran affairs or Medicare administrative claims data^[26–30]^ (n = 5), or surveys^[18,19]^ (n = 2). The proportion of patients receiving an OAC ranged from 9.7% in a study that used Medicaid data^[27]^ to 88.5% in a study that used a prospective registry of patients.^[24]^ Eight of the studies included patients on warfarin only, no studies included patients on DOACs only, and 5 studies included patients on either warfarin or DOACs. It was possible to report results by type of anticoagulant in one of the 5 studies that used warfarin or DOACs.^[25]^

**Table 1:** Impact of SDOH domains on anticoagulant prescription or use.

Study	OAC type	Exposure groups	Prescribed oral anticoagulant, N (%)	Adjusted OR (95% CI)
Education				
Chapman, 2017^[18]^ (N = 7,669,884)	Warfarin	*>*20% university graduates in patients zip code	672,572 (15.5%)	Reference
		<20% university graduates in patients zip code	678,692 (22.6%)	1.38 (1.38–1.38)
Goren, 2015^[19]^ (N = 1290)	Warfarin	<College	680 (52.7%)	Reference
		*>*Some college	610 (47.3%)	0.98 (0.68–1.42)
Employment				
Goren, 2015^[19]^ (N = 1290)	Warfarin	Unemployed	959 (74.3%)	Reference
		Employed	331 (25.7%)	0.64 (0.42–0.96)
Marital status				
Goren, 2015^[19]^ (N = 1,290)	Warfarin	Married	857 (66.4%)	Reference
		Single	87 (6.7%)	0.62 (0.31–1.23)
		Divorced/separated/widowed	346 (26.8%)	1.00 (0.67–1.49)
Socioeconomic status				
Chapman, 2017^[18]^ (N = 7,669,884)	Warfarin	>10% below federal poverty line	760,000 (20.9%)	Reference
		<10% below federal poverty line	599,645 (16.1%)	1.7 (1.7–1.7)
Goren, 2015^[19]^ (N = 1290)	Warfarin	<25,000	221 (17.1%)	Reference
		25,000–49,000	418 (32.4%)	1.84 (1.08–3.12)
		50,000–<75,000	270 (20.9%)	1.99 (1.09–3.64)
		75,000+	300 (23.3%)	1.90 (1.03–3.50)
		Declined to answer	81 (6.3%)	2.86 (1.24–6.60)
Smoking				
Goren, 2015^[19]^ (N = 1290)	Warfarin	Not current smoker	53 (17.5%)	Reference
		Current smoker	27 (7.9%)	0.68 (0.39–1.17)

### 3.3. Risk of bias in included studies

See Supplemental Digital Content 4, http://links.lww.com/MD/G992 for details on risk of bias in included studies. Ten of the included studies were not adjusted for all clinically important confounders (identified a priori for this review as age, sex, CHADS2 [or CHA2DS2-VASc], and bleeding risk) and were rated at high risk of bias due to confounding.^[18,19,21,22,25–30]^ Bleeding risk was the least frequent variable included in models. Selection bias was evaluated based on how patients were included into the study and risk of bias was low in all studies except for Goren, 2015 which included an online survey of patients who self-selected to participate. Patients who chose to participate and complete the survey may be more engaged in managing their disease and as a result more likely to receive guideline adherent treatment.^[19]^ Information bias was evaluated based on methods of outcome assessment, most of the studies used electronic health record or registry data were outcome data is extracted retrospectively irrespective of the exposure group, and were deemed at low risk of information bias, with the exception of 1 study where patients self-reported anticoagulant use.^[19]^ Methods of handling missing data were poorly described in 7 of the included studies and were deemed at high risk of bias due to missing data.^[21,22,25,26,28–30]^

### 3.4. Synthesis of results

#### 3.4.1. Gender and sexual orientation.

None of the studies reported on gender or sexual orientation.

Sex was evaluated in 9 studies (N = 7,783,260 patients)^[18–22,25,26,28]^ with a pooled OR of 1.08 (95% CI: 0.82, 1.43) indicating greater likelihood of receiving anticoagulant prescriptions among males compared to females. The pooled odds were not statistically significant. Heterogeneity of the pooled estimate was high (I^2^ = 99%). Subgroup analysis suggests that heterogeneity in the pooled estimate may partially be explained by type of anticoagulant used among patients who received a prescription. In studies that used warfarin only, the pooled odds of receiving anticoagulants was 1.17 (95% CI: 0.82, 1.67) indicating that males had a slightly greater likelihood of receiving anticoagulant prescriptions, although the difference was not statistically significant. In studies that used DOAC only, the pooled odds of receiving anticoagulants was 1.09 (95% CI: 1.00, 1.20) indicating that males had a slightly greater, and statistically significant, likelihood of receiving anticoagulant prescriptions. Test for subgroup differences *P* = .85; Fig. 1).

#### 3.4.2. Race/ethnicity, culture, religion, language, and immigration status.

**Figure 2.**
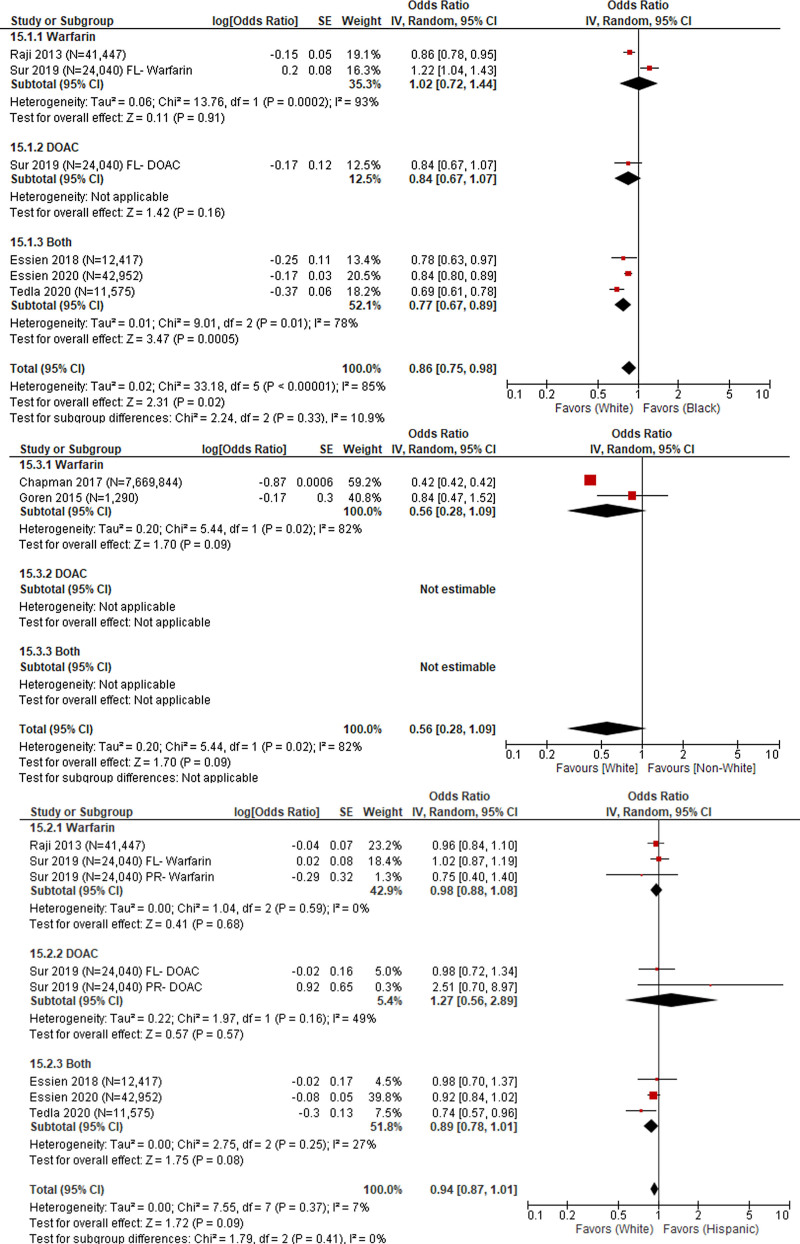
Race/ethnicity, culture, religion, language, and immigration status.

Ethnicity was evaluated in 5 studies (N = 132,431 patients)^[20,23–25,28]^ with a pooled OR of 0.94 (95% CI: 0.87, 1.01; Fig. 2) indicating a slightly lower likelihood of receiving anticoagulant prescriptions among Hispanic patients compared to non-Hispanic patients. The pooled odds were not statistically significant. Heterogeneity of the pooled estimate was low (*I*^2^ = 0%) and subgroup analyses were not conducted.

Black race was evaluated in 5 studies (N = 132,431 patients)^[20,23–25,28]^ with a pooled OR of 0.86 (95% CI: 0.75, 0.98) indicating a lower likelihood of receiving anticoagulant prescriptions among Black patients compared to White patients. Heterogeneity of the pooled estimate was high (*I*^2^ = 85%). Subgroup analysis suggested that heterogeneity in the pooled estimate may partially be explained by type of anticoagulant used among patients who received a prescription. Receiving anticoagulant prescription was similar by race in studies that reported using warfarin among patients who did receive anticoagulant prescription (OR: 1.02, 95% CI: 0.72, 1.44). Likelihood of receiving an anticoagulant prescription was lower among Black patients in studies that reported using DOAC (OR: 0.84, 95% CI: 0.67, 1.07; Test for subgroup differences *P* = .33; Fig. 2).

Non-White race was evaluated in 2 studies (N = 7,671,134 patients)^[18,19]^ with a pooled OR of 0.56 (95% CI: 0.28, 1.09; Fig. 2) indicating lower likelihood of receiving an anticoagulant prescription among non-White race compared to White race. The pooled odds were not statistically significant. Heterogeneity of the pooled estimate was high (*I*^2^ = 82%). The 2 studies evaluating non-White race used warfarin among patients who did receive anticoagulant prescription indicating that type of anticoagulant use did not contribute to the observed heterogeneity. Heterogeneity may partially be explained by methodological study characteristics, Goren et al^[19]^ included 1290 patients where data on atrial fibrillation diagnosis and anticoagulant use were self-reported through the US National Health and Wellness Survey. Chapman et al^[18]^ included a weighted sample of 7,669,844 patients from the 2010 National Ambulatory Care Survey (NAMCS) data which was extracted from patient medical records.

None of the studies evaluated associations with culture, religion, or language.

#### 3.4.3. Mental Health.

**Figure 3.**
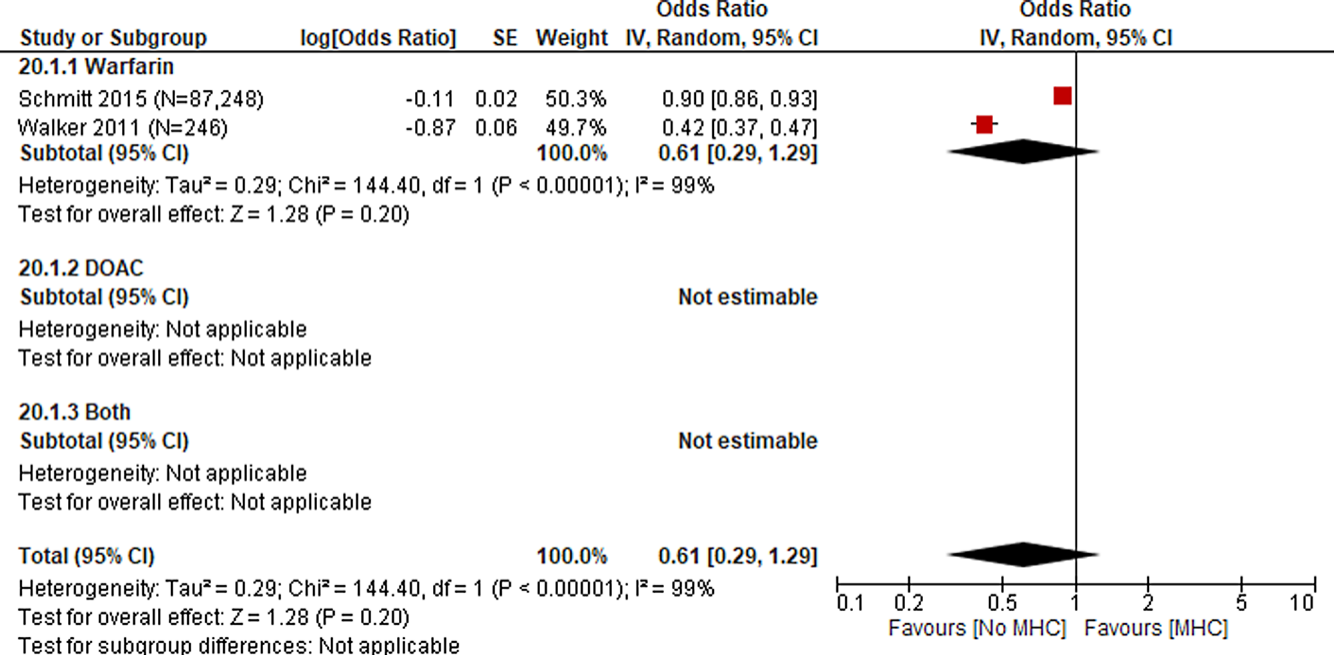
Mental health conditions.

Mental Health was evaluated in 2 studies (N=87,494)^[29,30]^ with a pooled OR of 0.61 (95% CI: 0.29, 1.29) indicating a lower likelihood of receiving an anticoagulant prescription among patients with mental health conditions compared to patients without mental health conditions. Heterogeneity of the pooled estimate was high (*I*^2^ = 99%). The 2 studies reported using warfarin among patients who did receive anticoagulant prescription indicating that type of anticoagulant use did not contribute to the observed heterogeneity (Fig. 3).

#### 3.4.4. Healthcare access and health literacy.

**Figure 4.**
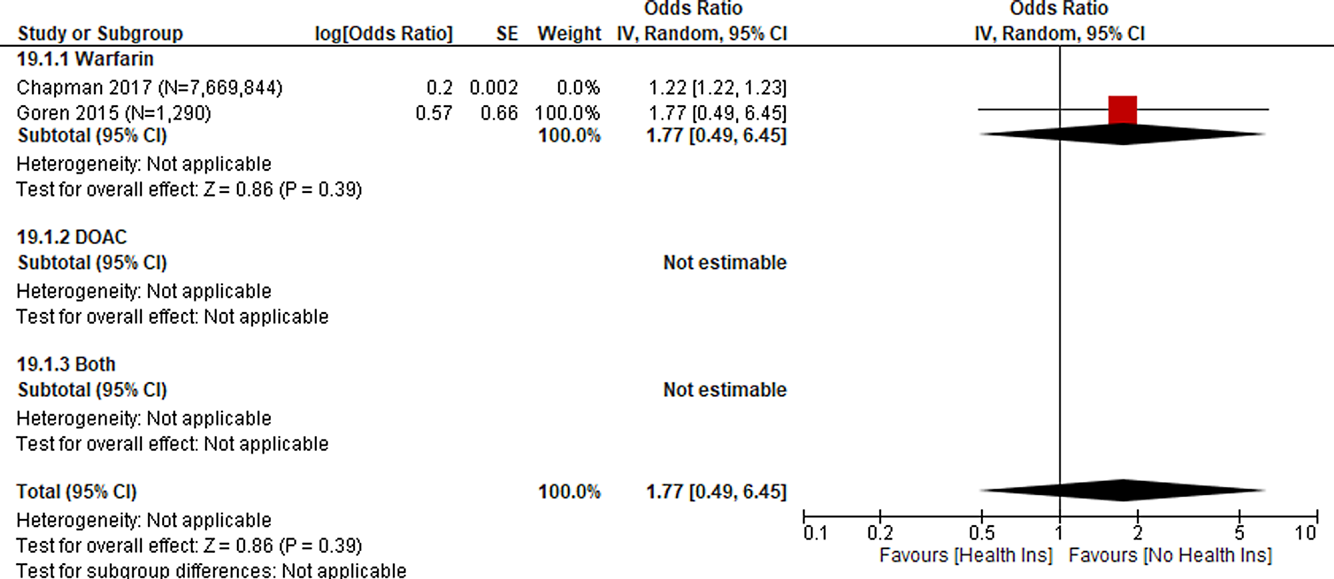
Health insurance.

Health insurance was used as a proxy for healthcare access and was evaluated in 2 studies (N = 7,671,134)^[18,19]^ with a pooled OR of 1.22 (1.22, 1.23) indicating a slightly greater likelihood of receiving anticoagulant prescription among patients without health insurance. Heterogeneity of the pooled estimate was low (*I^2^* = 0%). Both studies reported using warfarin among patients who received an anticoagulant prescription (Fig. 4).

None of the studies evaluated associations with other proxies for healthcare access or with health literacy.

#### 3.4.5. Health behaviors.

**Figure 5.**
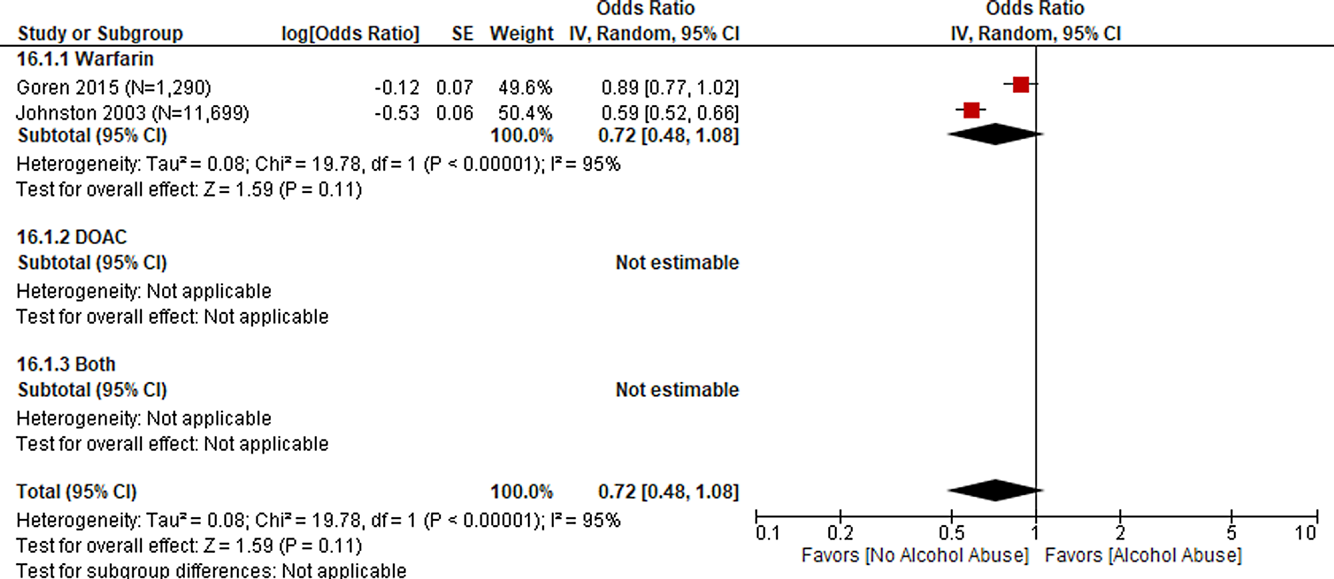
Impact of engaging in unhealthy behaviors on anticoagulant prescription or use.

Alcohol abuse was evaluated in 2 studies (N = 12,989 patients)^[19,27]^ with a pooled OR of 0.72 (95% CI: 0.48, 1.08) indicating a lower likelihood of receiving an anticoagulant prescription among patients who reported alcohol abuse compared to patients who did not report alcohol abuse. The pooled odds were not statistically significant. Heterogeneity of the pooled estimate was high (*I*^2^ = 95%). The 2 studies reported using warfarin among patients who received an anticoagulant prescription indicating that type of anticoagulant used did not contribute to the observed heterogeneity (Fig. 5).

Smoking was evaluated in 1 study (N = 1290 patients).^[19]^ A pooled OR was not feasible. The study reported an OR of 0.68 (95% CI: 0.39, 1.17) indicating lower likelihood of receiving anticoagulant prescription among patients who reported smoking compared to patients who did not report smoking. The study reported using warfarin among patients who received an anticoagulant prescription (Table 2). None of the studies evaluated other health behaviors including exercise or diet.

**Table 2:** Study characteristics.

Author, year	Data source	SDOH reported	Sample size	Type of OAC, N (%) study population on OAC	Outcomes reported and measurement definition	Age (y), mean + SD	% female	Variables adjusted for in multivariate models
Chae, 2011^[21]^	Electronic health record data	Sex	3086	Warfarin 2189 (71%) No OAC 897 (29%)	OAC prescription among patients with a CHADS_2_ = 1	OAC: 72 + 12 No OAC: 66 + 14	37%	Age, sex, nonparoxysmal AF, LV-dysfunction, coronary artery disease
Chapman, 2017^[18]^	National Ambulatory Medical Care Survey	Education, health insurance, race, SES, sex	7,669,844	Warfarin 1,372,476 (18%) No OAC 6,297,368 (82%)	OAC prescription	*>*75 y old: 4,125,244 (53.8%)	46%	Age, education, CHADS_2_ score, sex, race, health insurance status, percent poverty in patient zip code
Essien, 2018^[24]^	Prospective registry	Race	12,417	Warfarin 1704 (14%) DOAC 8791 (71%) No OAC 1922 (15%)	OAC prescription	White: 71 (64–78) Black: 67 (60–76) Hispanic: 72 (64–80)	41%	Household income by zip code level, education, insurance, US geographic location, demographics, medical history, medications, lab data, AF status, enrolling physician specialty
Essien, 2020^[20]^	Centers for Medicare and Medicaid Services Chronic Condition Data Warehouse	Race, sex	42,952	Warfarin 10,724 (25%) DOAC 10,537 (25%) No OAC 21,691 (50%)	OAC prescription after index AF diagnosis	Male: 79 + 10 Female: 74 + 10	42%	Age, sex, race/ethnicity, Medicaid eligibility, zip code-level median household income, CHA_2_DS_2_-VASc score, HAS-BLED, chronic kidney disease, recent history of bleeding, recent use of antiplatelets
Gage, 2000^[26]^	Medicare Part A claims	Sex	597	Warfarin 203 (34%) No OAC 394 (66%)	OAC prescription at discharge	>75 y old: 400 (67%)	55%	Age, sex, hospital location, prior embolic event, prior hemorrhage, blood dyscrasia, renal or hepatic disease
Goren, 2015^[19]^	National Health and Wellness Survey	Alcohol abuse, education, employment, health insurance, neighborhood, SES, smoking, social connections, sex	1290	Warfarin 542 (42%) No OAC 748 (58%)	OAC prescription, self-reported	VKA 68 + 10 ASA 65 + 10 VKA+ASA 66 + 12	35%	Age, sex, race/ethnicity, marital status, education, employment, health insurance, daily exercise, currently smoke, use alcohol, body mass index, income, CHADS_2_, comorbidity count
Johnston, 2003^[27]^	Ohio Department of Jobs and Family Services – Pharmacy, Medical, institutional claims	Alcohol abuse, race, sex	11,699	Warfarin 1136 (10%) No OAC 10,563 (90%)	OAC prescription filled 7 d before and 30 d after AF Dx	74 + 16	72%	Age, hypertension, congestive heart failure, prior hemorrhage (intracranial, gastrointestinal), predisposition to falls, alcohol or other drug abuse, perceived barriers to compliance, renal insufficiency
Kea, 2020^[22]^	Electronic health record data	Health insurance, race, sex	138	Warfarin 11 (8%) DOAC 9 (7%) No OAC 118 (85%)	OAC prescription at ED discharge	59 + 17	39%	Sex, CHA_2_DS_-_VASc stratification, cardiology consult
Raji, 2013^[28]^	Claims for a 5% national sample of Medicare beneficiaries	Race, sex	41,447	Warfarin 27,687 (67%) No OAC 13,760 (33%)	2+ OAC prescriptions filled on different dates in 2008	*>*75 y old: 30,104 (73%)	60%	Age, race, sex, census division, cardiologist visit, CHA_2_DS_2_-VASC score, medicaid eligibility, elixhauser comorbidity score
Schmitt, 2015^[29]^	Electronic health record data	MHC	87,248	Warfarin 45,498 (52%) No OAC 41,750 (48%)	OAC prescription	No MHC: 76 + 7 MHC: 73 + 9	No MHC: 1%MHC: 2%	Age, sex, race/ethnicity, CHADS_2_, physical comorbidity index
Sur 2019^[25]^	FL-PR CReSD Florida Puerto Rico Stroke Registry	Race, sex	24,040	Warfarin 7466 (31%) DOAC 4866 (20%) No OAC 11,708 (49%)	OAC prescription at discharge	79 + 11	54%	Age, sex, race-ethnicity, insurance status, academic hospitals, NIHSS, medical history of chronic renal insufficiency, length of stay, and serum creatinine
Tedla, 2020^[23]^	Electronic health record data	Race	11,575	Warfarin 3475 (30%) DOAC 2258 (20%) No OAC 5,842 (50%)	OAC within a year of AF diagnosis; type of OAC	73 + 12	54%	Age, sex, race, income, insurance status, CHA_2_DS_2_-VASc and HAS-BLED score
Walker, 2011^[30]^	VHA or Medicare outpatient and inpatient administrative data	MHC	246	Warfarin 162 (66%) No OAC 84 (44%)	OAC prescription	*>*75 y old No MHC: 115 (64%) MHC: 28 (43%)	No MHC: 3% MHC: 3%	Age and comorbidity index

AF = atrial fibrillation, MHC = mental health condition, OAC = oral anticoagulant, SES = socioeconomic status.

#### 3.4.6. Employment.

Employment status was evaluated in 1 study^[19]^ (N = 1290 patients). A pooled estimate was not feasible. The study reported an OR of 0.64 (95% CI: 0.42,0.96) indicating a lower likelihood of receiving an oral anticoagulant prescription among employed patients compared to unemployed patients.^[19]^ The study reported using warfarin among patients who did receive an anticoagulant prescription (Table 2).

#### 3.4.7. Socioeconomic status and financial strain.

Socioeconomic status was evaluated in 1 study (N = 7,669,844 patients).^[18]^ The study reported an OR of 1.7 (1.7–1.7) indicating a higher likelihood of receiving an oral anticoagulant among patients who live in a zip code where <10% of residents are below the federal poverty level compared to patients who live in a zip code where *>*10% of residents are below the federal poverty level.

Income level was evaluated in 1 study (N = 1290 patients).^[19]^ The study compared receiving an oral anticoagulant prescription across multiple income levels. Compared to patients reporting <$25,000 annually, patients who reported an income range of $25,000 to $49,000 reported an OR of 1.84 (1.08–3.12) indicating a higher likelihood of receiving an oral anticoagulant prescription. Results were similar when comparing patients reporting <$25,000 to higher income ranges ($50,000 to <$75,000 [OR: 1.99, 95% CI: 1.09, 3.64]; $75,000 or more [OR: 1.90, 95% CI: 1.03, 3.50]; declined to answer [OR: 2.86, 95% CI: 1.24, 6.60]). The study reported using warfarin among patients who did receive an anticoagulant prescription (Table 2).

#### 3.4.8. Community and social connections.

Marital status was evaluated in 1 study (N = 1290).^[19]^ A pooled estimated was not feasible. Compared to married patients, patients who reported being single had a 0.62 (0.31–1.23) lower likelihood of receiving an oral anticoagulant prescription. Compared to married patients, patients who reported being divorced, separated, or widowed were equally likely to receive oral anticoagulant prescription (OR: 1.00, 95% CI: 0.67, 1.49). The study reported using warfarin among patients who received an anticoagulant prescription (Table 2).

None of the studies evaluated other SDOHs related to community and social connections.

#### 3.4.9. Education.

Education status was evaluated in 2 studies (N = 7,671,134 patients). Education status definitions varied across the 2 studies and it was not feasible to provide a pooled estimate. The smaller study (N = 1290)^[19]^ showed no difference in anticoagulant use between the 2 education categories, defined as “less than college” versus “Some college or more” (OR: 0.98, 95% CI: 0.68, 1.42). The larger study (N = 7,669,884)^[18]^ categorized patients into education levels based on Zip code. Patients living in Zip Codes where <20% of residents are university graduates had a 1.38 (95% CI: 1.38, 1.38) greater likelihood of receiving oral anticoagulant prescription compared to patients living in Zip Codes where 20% or more of residents are university graduates. Both studies reported using warfarin among patients who did receive anticoagulant prescription (Table 2).

#### 3.4.10. Other SDOH domains.

The remaining SDOH domains were not evaluated in the studies included in this systematic review. These included disabilities, housing, food security, transportation, violence and interpersonal safety, neighborhood and built environment, and law and justice system.

## 4. Discussion

### 4.1. Summary of evidence

We systematically searched the literature to examine the impact of 16 SDOH domains identified a priori, by the US Preventive Service Task Force on anticoagulant prescriptions in patients with atrial fibrillation.^[12]^ The search identified 13 eligible studies that evaluated 9 SDOH. The number of studies evaluating each SDOH were small. Race and Ethnicity, which fall under the same SDOH domain, were the most evaluated, with 5 studies evaluating each. The remaining SDOH were evaluated in 3 or fewer studies only. Although heterogeneity was high, pooled estimates indicate that patients who report being Black or non-White were statistically significantly less likely to receive anticoagulants compared to non-Black patients or White patients, although the difference was statistically significant in the latter comparison only. Studies that used DOAC only, consistently showed lower odds of receiving treatment for Black patients,^[31]^ while results were mixed for studies that used warfarin only. The literature continues to report greater stroke incidence and mortality among Black patients with atrial fibrillation which can be partially explained by these findings.^[32]^

As expected, patients with mental illness were less likely to receive anticoagulant prescriptions. Similarly, patients reporting unhealthy behaviors, including alcohol abuse and smoking were less likely to receive anticoagulant prescriptions although estimates were not statistically significant. These results can be explained by the increased risk of bleeding in these patients, provider concerns of lack of ability to manage the treatment, and lack of social support.^[33,34]^ The included studies reporting on these SDOH domains used warfarin only. Further investigation is required to evaluate if these results are similar in DOAC which carries a smaller risk of bleeding and is easier to administer.

Despite the overwhelming evidence that anticoagulant use reduces the risk of stroke and mortality in patients with atrial fibrillation, prescription rates and use remain suboptimal.^[5]^ Results in this review are consistent with evidence in the literature highlighting the importance of evaluating SDOH in the management of atrial fibrillation.^[31,35]^ Our results confirm the need to increase national efforts in screening patients for social needs in the clinical setting and addressing their needs to support clinicians in providing guideline concordant care to their patients. The Institute of Medicine highlights the importance and need for evidence-based initiatives to better screen for and address social needs.^[9–11]^ More efforts need to be directed to implement these initiatives in every day clinical practice.

Data on cardiovascular health in people who are transgender and gender diverse is completely lacking from the literature.^[36]^ Despite the need, none of the included studies reported on the impact of gender and sexuality on anticoagulant use. Sex was not included in the list of SDOH used to create the search strategy.^[12]^ However, its impact was evaluated and reported in 8 of the included studies. The impact of sex was not statistically significant overall, although results trended to greater likelihood of prescriptions among males compared to females in studies using DOAC. This is consistent with the literature indicating lower use regardless of the levels of estimated thromboembolic risk.^[37–39]^ Potential reasons for this finding may be related to sex differences in acceptance of anticoagulant therapy. Other reasons may also include preconceived concerns in regard to bleeding risk among females.^[40,41]^

### 4.2. Limitations

We note a few limitations in this systematic review. Heterogeneity in the pooled estimates was high in most estimates. This was expected given the lack of consistent definitions and measures of SDOH. Included studies were limited to those conducted in the United States as an attempt to homogenize some of these SDOH. We employed a random effects model, determined a priori which involves an assumption that the effects being estimated in the different studies are not identical, but follow the same distribution.^[14]^ As an attempt to explain heterogeneity, we stratified by type of anticoagulant used (DOAC vs warfarin). Some studies used both and did not report results separately, so such stratification was not possible across all included studies. We are not able to stratify patients by thromboembolic risk; therefore, it is not possible to ascertain if the impact of SDOH is similar across different levels of risk. Finally, we focused this analysis on impact of SDOH on receiving an anticoagulant prescription. Further work is a need to evaluate the impact of SDOH on filling the prescription and on long-term adherence to anticoagulants overtime.

## 5. Conclusions

In conclusion, we comprehensively and systematically reviewed the literature to identify and quantify the impact of SDOH on receiving an anticoagulant prescription in patients with atrial fibrillation and describe 2 major findings. First, the literature reports on only half of the SDOH domains we searched for, indicating that many SDOH are not routinely assessed. Second, social needs impact the decision to prescribe anticoagulants, confirming the need to screen for and address social needs in the clinical setting in order support clinicians in providing guideline concordant care to their patients.

## Acknowledgments

We thank Ann Parks and Scott Glosner for their valuable feedback in developing the consent for this systematic review.

## Author contributions

Conceptualization: Khatib, Glowacki, Byrne, Brady

Data curation: Khatib, Glowacki, Byrne

Formal analysis: Khatib, Glowacki

Funding acquisition: Khatib

Investigation: Khatib, Glowacki, Byrne

Methodology: Khatib, Glowacki

Project administration: Khatib

Resources: Khatib, Glowacki

Software: Khatib, Glowacki

Supervision: Khatib, Brady

Validation: Khatib

Visualization: Khatib, Glowacki

Writing – original draft: Khatib, Glowacki

Writing: Khatib, Glowacki, Byrne, Brady

## Supplementary Material


