# Supplementary material for: Impact of social determinants of health on anticoagulant use among patients with atrial fibrillation: Systemic review and meta-analysis
Source: Medicine (Baltimore). 2022 Sep 2;101(35):e29997. doi: 10.1097/MD.0000000000029997 (PMC9439798; doi:10.1097/MD.0000000000029997)
Supplement: Supplementary file 1 [file medi-101-e29997-s001.pdf]

**Supplemental Material 1**. PubMed Search Algorithm			
Term Group	Grouping	Search Terms	# of Articles
Search Term	1	("Atrial Fibrillation"[TiAb]) OR "Atrial Fibrillation"[Mesh]	85,957
Search Term	2	(“Social Determinants of Health”[Mesh]) OR “Social Needs”) OR “Health Status Disparities”) OR “Socioeconomic Factors”) OR “race/ethnicity”) OR “tobacco use”) OR “alcohol use”) OR “residential address”) OR “education”) OR “financial resource strain”) OR “stress”) OR “depression”) OR “physical activity”) OR “social isolation”) OR “intimate partner violence”) OR “income”) OR “housing”) OR “food security”) OR “transportation”) OR “socioeconomic status”) OR “financial strain”) OR “violence”) OR “interpersonal safety”) OR “employment”) OR “community connections”) OR “social connections”) OR “Education”) OR “health behaviors”) OR “mental health”) OR “disabilities”) OR “neighborhood”) OR “build environment”) OR “culture”) OR “religion”) OR “language”) OR “Health care access”) OR “health literacy”) OR “law”) OR “justice system”) OR “gender”) OR “sexual orientation”) OR “domestic abuse”) OR “elder abuse”) OR “child maltreatment”) OR “substance use/abuse”) OR “physical activity”) OR “healthy diet”) OR “incarceration”) OR “prisoner”) OR “immigration”) OR “citizenship”	4,704,159
Search Term	3	("Anticoagulants"[Mesh]) OR "Stroke/prevention and control"[Mesh]) OR "Anticoagulation"[Tiab]) OR "Anticoagulants"[Tiab]) OR “Pescribing/prescription” [Tiab]) OR “Apixaban” [Tiab]) OR “ Rivaroxaban” [Tiab]) OR “Dabigatran” [Tiab]) OR “Edoxaban” [Tiab]) OR “Warfrin” [Tiab]	130,220
Total	4	1 AND 2 AND 3	1,537

**Supplemental Material 2**: Embase Algorithm			
Term Group	Grouping	Search Terms	# of Articles
Search Term	1	('Atrial Fibrillation':ab,ti OR 'Atrial Fibrillation'/de)	176,928
Search Term	2	('Social Determinants of Health'/exp OR 'Social Needs'/exp OR 'Health Status Disparities' OR 'Socioeconomic Factors' OR 'race/ethnicity' OR 'tobacco use' OR 'alcohol use' OR 'residential address' OR 'education' OR 'financial resource strain' OR 'stress' OR 'depression' OR 'physical activity' OR 'social isolation' OR 'intimate partner violence' OR 'income' OR 'housing' OR 'food security' OR 'transportation' OR 'socioeconomic status' OR 'financial strain' OR 'violence' OR 'interpersonal safety' OR 'employment' OR 'community connections' OR 'social connections' OR 'Education' OR 'health behaviors' OR 'mental health' OR 'disabilities' OR 'neighborhood' OR 'build environment' OR 'culture' OR 'religion' OR 'language' OR 'Health care access' OR 'health literacy' OR 'law' OR 'justice system' OR 'gender' OR 'sexual orientation' OR 'domestic abuse' OR 'elder abuse' OR 'child maltreatment' OR 'substance use/abuse' OR 'physical activity' OR 'healthy diet' OR ‘incarceration’ OR ‘prisoner’ OR ‘immigration’ OR ‘citizenship’)	7,034,925
Search Term	3	('Anticoagulants'/de OR 'Stroke/prevention and control'/de OR 'Anticoagulation':ab,ti OR 'Anticoagulants':ab,ti OR ‘Prescrbing/prescription’:ab,ti OR ‘Apixaban’:ab,ti OR ‘Rivaroxaban’:ab,ti OR ‘Dabigatran’:ab,ti OR ‘Edoxaban’:ab,ti OR ‘Warfarin’:ab,ti)	208,257
Search Term	4	([embase]/lim NOT ([embase]/lim AND [medline]/lim)	10,132,290
Total	5	1 AND 2 AND 3 AND 4	2,478

Supplemental Material 3: PRISMA Flow Diagram

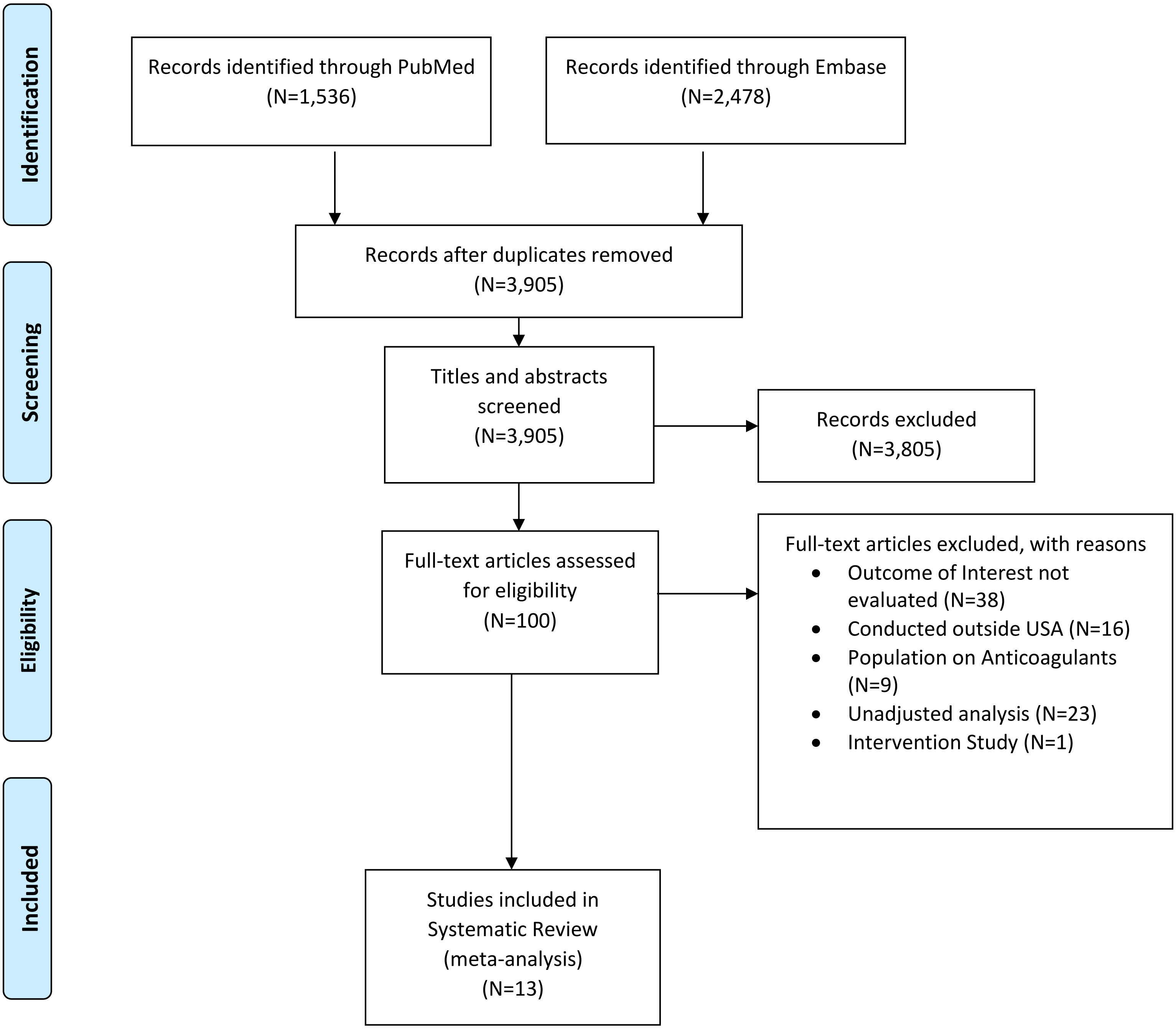

Supplemental Material 4: Risk of bias across individual studies

Author, year	Bias due to confounding (were important confounding variables* adjusted for?)	Selection bias (was selection into the study unrelated to exposures and outcomes?)	Information bias (were methods of outcome assessment comparable across exposure groups?)	Bias due to missing data (were reasons for missing data unrelated to exposure and outcomes?)
Chae, 2011	High Risk	Low Risk	Low Risk	High Risk
Chapman, 2017	High Risk	Low Risk	Low Risk	Low Risk
Essien, 2018	Low Risk	Low Risk	Low Risk	Low Risk
Essien, 2020	Low Risk	Low Risk	Low Risk	Low Risk
Gage, 2000	High Risk	Low Risk	Low Risk	High Risk
Goren, 2015	High Risk	High Risk	High Risk	Low Risk
Johnston, 2003	High Risk	Low Risk	Low Risk	Low Risk
Kea, 2020	High Risk	Low Risk	Low Risk	High Risk
Raji, 2013	High Risk	Low Risk	Low Risk	High Risk
Schmitt, 2019	High Risk	Low Risk	Low Risk	High Risk
Sur, 2019	High Risk	Low Risk	Low Risk	High Risk
Tedla, 2020	Low Risk	Low Risk	Low Risk	Low Risk
Walker, 2011	High Risk	Low Risk	Low Risk	High Risk

*Estimates adjusted for at least age, sex, CHADSVASC, and bleeding risk were considered low risk
